# Key design considerations for adaptive clinical trials: a primer for clinicians

**DOI:** 10.1136/bmj.k698

**Published:** 2018-03-08

**Authors:** Kristian Thorlund, Jonas Haggstrom, Jay JH Park, Edward J Mills

**Affiliations:** 1 Department of Health Research Methods, Evidence, and Impact (HEI), McMaster University, Ontario, Canada; 2 The Bill and Melinda Gates Foundation, Seattle, Washington, USA

## Abstract

This article reviews important considerations for researchers who are designing adaptive clinical trials. These differ from conventional clinical trials because they allow and even enforce continual modifications to key components of trial design while data are being collected. This innovative approach has the potential to reduce resource use, decrease time to trial completion, limit allocation of participants to inferior interventions, and improve the likelihood that trial results will be scientifically or clinically relevant. Adaptive designs have mostly been used in trials evaluating drugs, but their use is spreading. The US Food and Drug Administration recently issued guidance on adaptive trial designs, which highlighted general principles and different types of adaptive clinical trials but did not provide concrete guidance about important considerations in designing such trials. Decisions to adapt a trial are not arbitrary; they are based on decision rules that have been rigorously examined via statistical simulations before the first trial participant is enrolled. The authors review important characteristics of adaptive trials and common types of study modifications and provide a practical guide, illustrated with a case study, to aid investigators who are planning an adaptive clinical trial

**Summary points**

Adaptive trials enable continual modification to the trial design based on interim data. They can reduce use of resources and time or improve the likelihood of success of the trial
Common adaptive designs allow for interim sample size reassessment to ensure sufficient power, adaptation of the allocation ratio to ensure more patients receive the superior treatment, dropping of inferior treatments, addition of new treatment arms to save time and resources, population “enrichment” to narrow scope of the clinical trial, or transition directly from one trial phase to another
Planning adaptive trials is rooted in comprehensive simulations to understand the likely consequences and gains of all possible adaptations and the appropriateness of the incorporated decision rules. Simulations should be planned with clinical input and be transparent
Statistical analysis plans for adaptive trials should cover interim analyses to optimise efficiency and final analyses to draw final conclusions about the observed treatment differences

Adaptive clinical trials can be completed sooner than trials with conventional (non-adaptive) designs. The US Food and Drug Administration (FDA) and the European Medicines Agency (EMA) have recently released guidance on adaptive designs for licensing.^1^ ^2^ But little guidance exists on how investigators should proceed when designing and planning an adaptive clinical trial. We outline and discuss common characteristics and study modifications of adaptive trials and provide a practical planning guide for designing and interpreting adaptive clinical trials.

## Characteristics of adaptive designs

Adaptive designs allow for modifications to key components. Unlike conventional designs, where the learning typically occurs after the trial is completed, adaptive designs intend for continual learning as the data accumulate. Several characteristics are more common in, or unique to, adaptive trials than conventional trials (box 1). Changes can be made to the allocation ratio, total sample size, and eligibility criteria, trials can be extended from phase II into phase III, or treatment arms can be added or dropped. Adaptive trials have the potential for decreased time to completion, reduced resource requirements and number of patients exposed to inferior treatments, and overall improved likelihood of trial success. But they also come with the risk of creating inefficiencies if poorly planned. Any possible decision for adaptation should undergo rigorous risk-benefit assessment, such that the potential scientific and ethical gains outweigh the risks of causing bias or trial inefficiencies.

Box 1 **What makes a randomised clinical trial adaptive?**

Key study design components can be adapted throughout the trial
Trial planning involves several rounds of simulations
Consequences and gains of possible trial adaptations need to be understood before initiation
Statistical analysis plans are needed for both interim and final analyses
Research question may change along with adaptations (for example, narrowing the population)
Multiple trials (such as phase II and III) can seamlessly be combined in one adaptive trial
New experimental treatments can be added rather than starting a new separate trial

## Common types of adaptive trials

**Fig 1:**
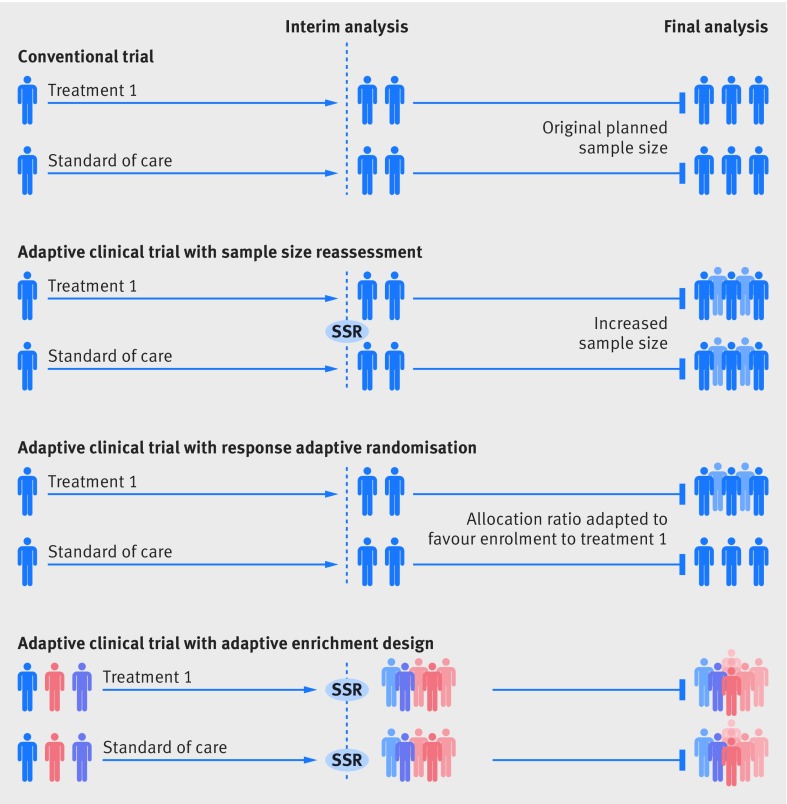
Common types of adaptive trials. Sample size reassessment: if interim analysis shows worse results than expected, the sample size can be reassessed and increased to ensure that the trial is adequately powered. Response adaptive randomisation: if interim analysis shows promising results for a treatment, the allocation ratio can be modified to favours enrolment to that treatment. Adaptive enrichment: if interim analysis shows that a treatment has more promising results in one subgroup of patients, the study eligibility criteria can be modified to investigate the efficacy of the intervention in the that subgroup, with a sample size reassessment to ensure a sufficient sample size. SSR=sample size reassessment

Common types of adaptive clinical trials include, but are not limited to, sample size reassessment,^2^ ^3^ response adaptive randomisation and dropping of inferior treatment arms,^4^ adaptive enrichment,^5^ and “seamless” designs (fig 1). Sample size reassessment uses event based evaluations during the trial to determine actual power.^3^ ^6^ Response adaptive randomisation allows for changes in the randomisation ratio during the trial, so that, if interim results are favourable, newly enrolled patients are more likely to be assigned to the treatment arm.^4^ Adaptive enrichment refers to a modification to the trial eligibility criteria or outcome evaluations; if interim analysis shows that one subgroup has a more favourable response, the trial can be “enriched” by modifying its eligibility criteria to either solely or predominantly enrol patients from this subgroup.^5^ Similarly, clinical and biochemical outcomes may be augmented to enhance the trial’s relevance, wide application, or probability of success. Seamless adaptive trial designs permit continuation from one phase to the subsequent phase, generally from phase II to phase III trials. The results from the phase II trial can be used to determine the initial allocation ratio, the planned total sample size, and a potentially enriched set of population for the subsequent phase III.

## Planning an adaptive trial with realistic expectations

Some conventional analyses have interim analyses, which typically use prespecified rules for early termination (such as O’Brien-Fleming monitoring boundaries) that are not adaptive.^7^ But all adaptive trials have interim analyses with the possibility for design adaptations, making their planning more extensive. Investigators should consider and anticipate challenges associated with all possible trajectories of adaptations and should establish decision rules that minimise the risk of biased or inefficient adaptations. They must perform extensive trial simulations on multiple scenarios for the risk-benefit assessment.

**Fig 2:**
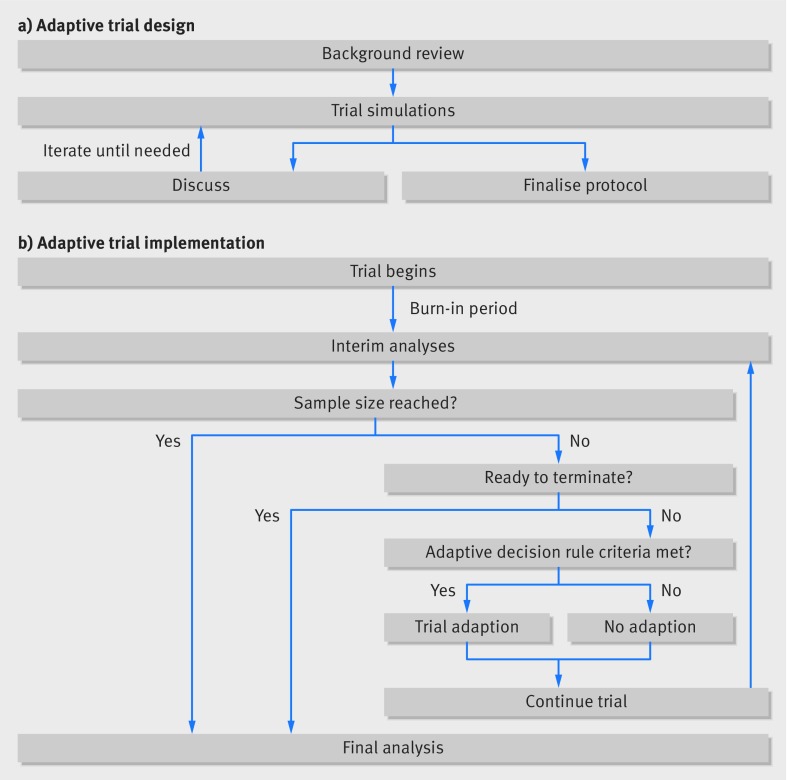
Adaptive design planning process

**Fig 3:**
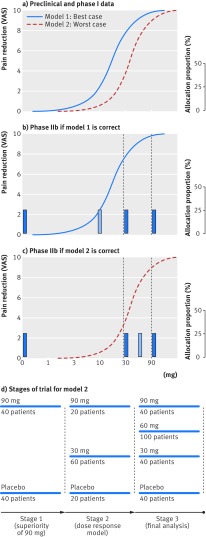
Case study of a dose-response adaptive trial. VAS=visual analogue scale

The adaptive design is heavily rooted in simulations (fig 2). These are expanded on and re-run until investigators and trial statisticians are confident that the likely benefits of the adaptive design substantially outweigh the potential risks. After establishing a sufficiently robust design, they can finalise the trial protocol and start the trial. Implementing adaptive trials commonly involves a cycle of interim analyses and decisions (fig 2b). A practical case study of an adaptive clinical trial is presented in box 2 and fig 3.

Box 2 **Case study of a dose-response adaptive trial**

A drug for knee pain showed an effect at roughly 10 mg in a preclinical model (model 1), and an effect between 30 and 90 mg in pharmacokinetic and pharmacodynamic models from phase I data (model 2; fig 3a). The aim of this trial was to first establish superior efficacy of 90 mg over placebo (phase IIa) and to find the median effective dose (ED50) using dose-response modelling (phase IIb).

Given budget and time constraints corresponding to enrolment of 400 patients, preliminary simulations had shown that if the assumed model (either model 1 or model 2) was correct, four treatment arms with equal allocation would be optimal to establish the ED50 and dose-response model. But an incorrect assumption about the dose-response could lead to inefficiencies. The manufacturer was interested in testing either 0, 10, 30, and 90 mg or 0, 30, 60, and 90 mg, depending on which model was the most accurate.

Given the expected efficacy from phase I evidence (model 2), our sample size calculation showed that this could be achieved at a 20% one-sided alpha level with 40 patients to each arm. So the first interim analysis was planned after 80 patients with a decision-rule of stopping the trial if 90 mg did not show an effect at this time.

Phase IIb needed to tell us which two of the three remaining doses should be used. If model 1 was the most accurate, then efficacy increments would predominantly occur between 0 and 30 mg, so enrolling patients to 10 mg and 30 mg would be optimal for estimating dose-response (fig 3b). If efficacy increments predominantly occurred between 30 and 90 mg (model 2, enrolling patients to 30 mg and 60 mg would be optimal (fig 3c). Both models have 30 mg in common, so the second stage was designed to randomise an additional 100 patients in a ratio of 1:3:1 to receive 0, 30, and 90 mg, respectively. The decision rule implemented at the end of this stage was to pick 10 mg or 60 mg as a fourth arm depending on whether the efficacy of 30 mg efficacy was <50% of the 90 mg efficacy.

At the start of the third stage a total of 180 patients had been randomised, with 60 patients randomised to each of the three arms (0 mg, 30 mg, and 90 mg). As the trial aim was to randomise 100 patients to each of the four final arms, an additional 40 patients would be randomised to the existing three arms and a 100 patients would be randomised to the fourth arm (either 10 mg or 60 mg).’Thus, at the third stage patients were randomised at 2:5:2:2 for model 1 or 2:2:5:2 for model 2 (fig 3d). The final analysis of 400 patients, as per preliminary simulations, be powered by >80% to establish the median effective dose.

## Clinical trial simulations

Simulations can be used for any type of study design but are customarily used in adaptive designs owing to the multitude of trajectories. They are used to establish statistical and practical properties of adaptive trial designs. The risks of false positives (type I error) and false negatives (type II error) in adaptive trials are difficult (if not impossible) to evaluate with conventional methods.^6^ Regulatory agencies commonly require control of these errors, so they should be decision-rules, using metrics such as expected reduction in required sample size, time to completion, number of treatment failures avoided, risk of biased interim effect estimates, and robustness of the planned statistical analysis at trial termination. These can be particularly helpful for planning a realistic budget and timeline.

Because simulation is an iterative process (fig 2), we recommend starting with few^2^ ^3^ “best case” and “worst case” scenarios based on current evidence or opinion, such as the two dose-response models in the case study (box 2). Exploring several scenarios can help quickly establish the likely efficiency gain from applying an adaptive design over a conventional design. Further, starting group discussions with only simple simulations can be important for engaging the clinical expertise of the investigators and help them to become more familiar with interpreting trial simulations. Then more comprehensive simulations extended to other scenarios can be shared for feedback. Statisticians should be engaged early in the planning phase. Trial simulations can be time consuming. Regulatory agencies should also be involved early in the planning phase.

## Decision rules

Decision rules are prespecified before starting the trial. The most common types are mathematical expressions for terminating treatment arms or the trial and modifications of the allocation ratio between treatments. Other decision rules include preset quantitative criteria for re-estimating the sample size or narrowing patient eligibility or picking new arms to best inform a dose-response model (box 2).

Investigators must also consider which outcomes will be evaluated for decision rules and what effect the choice of outcome has on the properties of the design; for example, risk of false positive “enrichment.” To determine the adequacy of the outcomes used for the decision rules, the clinical relevance and time required to measure the outcome are essential. The outcome for the decision rule should be clinically important and sufficiently correlated with at least one of the trial’s primary outcomes. If the outcome fails to meet these criteria, the clinical merit and statistical robustness of any trial adaptation is likely to be inadequate. The time required to observe the outcome, and therefore collect sufficient interim data, should be short enough that efficiency, ethical gains, or both can be achieved with adaptations before the trial is terminated. For example, if one year progression free survival is used to inform response adaptive randomisation in a trial that enrols patients over two years, the time required to collect, clean, and analyse enough information to support an interim trial adaptation is so substantial that only a small proportion (the last 25%) of enrolled patients may benefit from this adaptation and no real efficiency gains will be realised. By contrast, an outcome such as three month tumour response may facilitate the collection of sufficient data to inform early adaptations but may lack sufficient correlation with the primary outcome (such as full trial duration overall survival). Six month progression free survival may strike a more appropriate balance between time to collect and robustness.

Transparency is key to decision rules and adaptive trial designs. The outcome used as a decision rule to inform an adaptation should be clear, as should the justification for the outcome and the decision rules. Moreover, decision rules should be easy to implement and implement, to avoid practical adaptation that may impose bias. If the trial is planned for regulatory approval, further considerations should ensure that the decision rules are also non-binding. That is, whether a decision rule is enforced should not affect statistical properties such as type I error. Multiple sophisticated statistical and economic decision rules to accommodate optimal adaptations have been described in detail elsewhere.^8^ ^9^

## Statistical planning

Statistical planning is critical for any clinical trial design. For adaptive designs, the statistical analysis plan comprises the simulation, the interim analysis to inform potential adaptations, and the final analysis of the completed trial. Similar to conventional trial designs, a number of factors such as observed and expected effects, trial budget, and total maximum sample size need to be considered.

Adaptive trials need a so-called burn-in phase where a predetermined number of patients can be enrolled at a fixed allocation ratio (usually 1:1) to ensure that enough data are collected to allow for reasonable expected precision. Making adaptations too early can be problematic because small datasets are more prone to random error.

The general rule of thumb for response adaptive randomisation is to collect data on a minimum of 20-30 patients in each arm (the burn-in) before conducting the first interim analysis.^10^ Other adaptations, including sample size reassessments and study enrichments, typically need a longer burn-in period and larger sample size.

In multi-arm adaptive trials, allocation to the control group is commonly fixed (for example, at 20% of total patient allocation) and the allocation ratio between experimental treatments is adjusted.^11^ ^12^ Collecting sufficient data from the control group helps to ensure adequate statistical power to make comparisons between treatments.

The final statistical analysis of an adaptive trial is usually quite similar to that of conventional trials. This may be because regulatory requirements commonly have heavy emphasis on conventional statistics (such as requiring P values less than 5%). But using more specific statistics when making the decision to adapt a trial during interim analyses may be the cause of misinterpretation if viewed through the lens of conventional statistics. For example, if an adaptive multi-arm trial allows early termination due to a predefined threshold for a (bayesian) probability of treatment superiority, a conventional statistical analysis of the final dataset may not meet regulatory success criteria. In particular, a two sided statistical test comparing the apparently superior treatment to the control could yield a P value larger than 5%, which is very different from estimating the bayesian probability of superiority among many treatments, especially when data are still relatively small during an interim analysis. Like any clinical trial stopped early, adaptive trials may also yield treatment effect estimates that are affected by random error. Planning which types of statistical inferences will be drawn in case of early termination is important. For example, a seamless adaptive trial may reliably inform which of many candidate treatments (or doses) tested in phase II should be continued into a phase III, but claims about the magnitude of treatment effects may not be warranted.

## Concluding remarks

Adaptive randomised trials offer several advantages over conventional randomised trials. But the efforts and time required for planning and implementing adaptive trials are typically more exhaustive and do not always offer important gains in efficiency or ethics. This is a practical guide for future investigators to design an adaptive clinical trial. Understanding the use and merit of trial simulations during the planning stage, how to determine the adequacy of decision rules for trial adaptations, as well as unique aspects of the statistical planning is key to the success of adaptive clinical trials.

## References

[1] European Medicines Agency. Adaptive pathways 2017 http://www.ema.europa.eu/ema/index.jsp?curl=pages/regulation/general/general_content_000601.jsp.

[2] United States Food and Drug Administration. Adaptive designs for medical device clinical studies., 2016. https://www.fda.gov/downloads/medicaldevices/deviceregulationandguidance/guidancedocuments/ucm446729.pdf

[3] Guyatt GH Mills EJ Elbourne D In the era of systematic reviews, does the size of an individual trial still matter. PLoS Med 2008;5:e4. 10.1371/journal.pmed.0050004 18177203 PMC2174963

[4] Bauer P Koenig F The reassessment of trial perspectives from interim data--a critical view. Stat Med 2006;25:23-36. 10.1002/sim.2180 16220517

[5] Ning J Huang X Response-adaptive randomization for clinical trials with adjustment for covariate imbalance. Stat Med 2010;29:1761-8. 10.1002/sim.3978 20658546 PMC2911996

[6] Simon N Simon R Adaptive enrichment designs for clinical trials. Biostatistics 2013;14:613-25. 10.1093/biostatistics/kxt010 23525452 PMC3769998

[7] O’Brien PC Fleming TR A multiple testing procedure for clinical trials. Biometrics 1979;35:549-56. 10.2307/2530245 497341

[8] Hummel J Wang S Kirkpatrick J Using simulation to optimize adaptive trial designs: applications in learning and confirmatory phase trials. Clin Investig (Lond) 2015;5:401-13 10.4155/cli.15.14.

[9] Antonijevic Z Kimber M Manner K Burman C Pinheiro J Bergenheim K Optimizing Drug Development Programs. Ther Innov Regul Sci 2013;47:7 10.1177/2168479013480501. 30231431

[10] Bornkamp B Bretz F Dmitrienko A Innovative approaches for designing and analyzing adaptive dose-ranging trials. J Biopharm Stat 2007;17:965-95. 10.1080/10543400701643848 18027208

[11] Atkinson AC Biswas A Randomised response-adaptive designs in clinical trials. CRC Press, 2013.

[12] Wason JM Abraham JE Baird RD A Bayesian adaptive design for biomarker trials with linked treatments. Br J Cancer 2015;113:699-705. 10.1038/bjc.2015.278 26263479 PMC4559835

